# Cellular senescence as a therapeutic target for aging intervention

**DOI:** 10.1016/j.bj.2026.100948

**Published:** 2026-01-17

**Authors:** Youkun Bi, Guangju Ji

**Affiliations:** a Henan Academy of Sciences, Zhengzhou, China; b State Key Laboratory of Epigenetic Regulation and Intervention, Institute of Biophysics, Chinese Academy of Sciences, Beijing, China

**Keywords:** Cellular senescence, Senolytics, Senomorphics, Senescence immunotherapy, Restoration-oriented interventions

## Abstract

Cellular senescence is a stress-induced cellular state that contributes to tissue dysfunction, chronic inflammation, and a broad range of aging-associated pathologies. The accumulation of senescent cells (SnCs) disrupt normal tissue function, positioning them as drivers of pathological decline and therapeutic targets for aging intervention. Accordingly, multiple senescence-targeted strategies have been developed, including senolytics, senomorphics, senescence immunotherapy, and restoration-oriented interventions. These approaches aim to mitigate senescence-driven pathology by eliminating senescent cells, modulating their secretory activity, or restoring cellular function. Ongoing advancements will require precise stratification of senescent states, careful assessment of long-term safety, and the integration of optimized delivery systems for targeted therapeutic outcomes.

## Highlights

• Senescent cells are closely associated with aging and aging-related disorders.
• Senotherapeutics comprise senolytics, senomorphics, senescence immunotherapy, and restoration-oriented interventions.
• Senoreverse highlights pluripotency-independent interventions to rejuvenate senescent cells and preserve lineage identity.

## 1 Introduction

Aging is a complex and multifactorial biological process that has been framed within conceptual models, most notably the canonical hallmarks of aging, encompassing cellular senescence, genomic instability, telomere attrition, epigenetic alterations, mitochondrial dysfunction, stem cell exhaustion, and altered intercellular communication [1,2]. However, there remains no broad consensus on the fundamental definition of aging, its primary causes, or the existence of a single molecular or cellular process as a central determinant of aging [3,4]. Specifically, these divergent perspectives emphasize on distinct aspects of aging, including damage accumulation, functional decline, systemic dysregulation, or emergent organism-level properties [5,6].

**Fig. 1:**
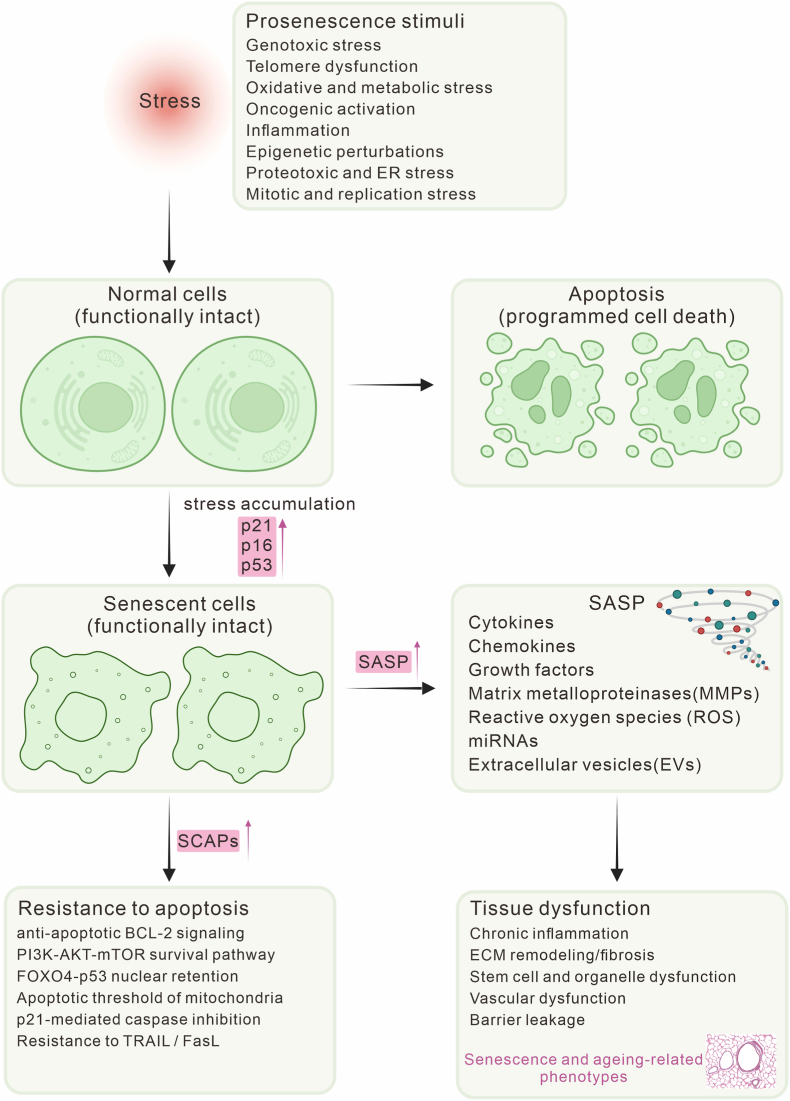
Cellular stress and senescence phenotypes. Diverse cellular stresses drive cells toward apoptosis or senescence. SnCs undergo durable growth arrest mediated by the p53-p21 and p16-RB pathways, acquire resistance to apoptosis through SCAPs, and develop SASP that releases cytokines, proteases, reactive oxygen species, and extracellular vesicles. Collectively, these features promote chronic inflammation, extracellular matrix(ECM) remodeling, stem-cell dysfunction, and vascular decline, thereby linking senescence to tissue dysfunction and age-related disease.

Senescence is a stress-induced cellular state that is classically associated with durable growth arrest and, through the senescence-associated secretory phenotype(SASP), can propagate inflammation and tissue dysfunction [2,7]. Importantly, many senescent cells persist because they acquire heightened resistance to apoptosis and become dependent on senescence-associated anti-apoptotic pathways(SCAPs) (Fig. 1). While senescence serves essential roles in early life, including tissue remodeling and tumor suppression, its age-related accumulation and persistence can erode tissue function and accelerate pathologies such as neurodegeneration, fibrosis, and metabolic decline [8,9]. Given its central role, targeting senescent cells has emerged as a promising therapeutic strategy to mitigate aging and its associated disorders [10,11].

In this review, we outline core principles governing cellular senescence and its diverse manifestations across tissues and cell types. Major senescence-targeted strategies are then discussed, along with key considerations for their translational application in age-associated diseases.

## 2 Defining and contextualizing cellular senescence

### 2.1 Definition and core features of cellular senescence

On the basis of current evidence, cellular senescence can be defined as a stress-evoked, durable cellular state stabilized by persistent engagement of damage-response and tumor-suppressor programs [2]. This stabilization is typically accompanied by long-term reconfiguration of chromatin, gene regulation, and metabolism, leading to constrained cellular competence and remodeled intercellular signaling [12,13]. In proliferative lineages, senescence is evidenced by durable loss of proliferative capacity, which is typically enforced by sustained activation of the p53-p21^WAF1/Cip1^ and p16^INK4a^-RB axes [14,15]. In post-mitotic lineages, where proliferative arrest is non-informative, senescence is inferred from persistent stress signalling with chromatin and transcriptional rewiring, organellar dysfunction, and sustained SASP-like outputs that propagate non-cell-autonomous effects [16,17]. Accordingly, senescence should be assigned only when phenotypic persistence is demonstrated independently of continued stimulus and multiple orthogonal features converge within the same cells.

Translating this definition into practice requires criteria for assigning senescence within intact tissues, as no single marker is sufficiently specific [1]. Senescence is therefore assigned when multiple orthogonal hallmarks converge within the same cells and persist over time, rather than reflecting a transient stress response. This convergence is best supported by concordant evidence from complementary readouts, for example cell-cycle inhibition (*CDKN1A*/*CDKN2A* induction with reduced *MKI67*) [18], persistent DNA damage response signals (γH2AX/TP53BP1 foci) [5], lysosomal expansion (SA-β-gal activity) [15], and nuclear reorganization (LMNB1 loss) [19]. SASP secretory outputs frequently represent the most functionally consequential feature, linking SnCs to chronic inflammation and microenvironmental remodeling. However, SASP are highly cell type- and tissue-dependent, and the absence of a limited set of canonical SASP factors should not be used to rule out senescence [15,20]. When feasible, high-plex transcriptomic and proteomic profiling can more faithfully capture SASP heterogeneity and mitigate misclassification driven by any single readout [21,22].

SASP comprises a broad, dynamically regulated repertoire of soluble and vesicle-associated factors, including inflammatory cytokines and chemokines, growth factors, proteases, and extracellular matrix [20,23]. Rather than a fixed signature, SASP is modular and time-evolving, shaped by cell identity, initiating stress, and the regulatory circuitry that couples persistent stress signals to transcriptional regulation, for example, DDR-linked NF-κB and C/EBPβ programmes, p38-mTOR signalling, and innate immune sensing such as cGAS-STING [24]. Functionally, the SASP serves as a central mediator of senescence-associated non-cell-autonomous effects, reinforcing senescence through autocrine signalling, propagating paracrine senescence or stem-cell suppression, remodeling extracellular matrices, and recruiting immune effectors that mediate surveillance and clearance [25]. Moreover, a transient and spatially constrained SASP can support tissue repair and reinforce tumour-suppressive programmes [26]. By contrast, a persistent and self-sustaining SASP can drive chronic inflammation, fibrosis, and tissue dysfunction, positioning SASP as both a central determinant of senescent biology and a therapeutically actionable, yet context-sensitive, target [4].

Together, these features provide a pragmatic framework for identifying and studying cellular senescence across biological systems, accommodating the intrinsic diversity of senescent states and laying the conceptual groundwork for subsequent discussion of senescence heterogeneity and therapeutic targeting.

### 2.2 Heterogeneity and determinants of senescent states

Single-cell and spatially resolved analyses of intact tissues indicate that senescence is organized as a modular programme with a relatively conserved stress-arrest core coupled to variable effector modules that shape tissue-level outcomes [7,27]. A key determinant of variation among senescent states is their effector wiring. In some, cytokine and chemokine production dominate, driving inflammatory responses [28], while others activate TGF-β-dependent extracellular matrix remodeling that contribute to fibrosis [29]. Additionally, certain senescent states exhibit a bias toward innate immune activation or interferon-like responses, such as the cGAS-STING axis [30]. Commonly used in vivo markers can also map onto partially distinct senescent subpopulations, For example, p16^INK4a^ and p21^WAF1/Cip1^ label overlapping yet non-identical populations with distinct secretory outputs and, in functional models, divergent contributions to pathology [31]. Together, these features explain why senescence signatures often transfer poorly across organs and why interventions should be matched to the dominant effector module and tissue liability rather than treating senescence as a unitary target [21].

The determinants of these effector modules lie in how distinct initiating lesions are sensed and coupled to transcriptional control, and this coupling can evolve as senescence persists [32]. DDR- and p38-mTOR-linked signalling often engages NF-κB and C/EBPβ to drive inflammatory outputs [33], whereas alternative wiring can route the same core state toward TGF-β-associated extracellular-matrix programmes [29]. Mitochondrial dysfunction illustrates that durable stress-arrest decouple from canonical inflammatory secretion, producing a stable arrest state with selective attenuation of IL-1-dependent inflammatory outputs while preserving non-inflammatory programmes that reshape differentiation and tissue function [34]. In parallel, mislocalized self nucleic acids add a further axis of diversification, as persistent cytosolic DNA can activate cGAS-STING and superimpose interferon- and ISG-biased transcriptional outputs onto the senescent core [30,35]. These driver-dependent couplings are further shaped by lineage-specific regulatory landscapes and by niche and immune constraints, helping to explain why comparable senescent burdens may resolve in one tissue yet persist as chronic inflammation or fibrosis in another.

Such heterogeneity has direct in vivo consequences, because marker-defined senescent subsets are not interchangeable and respond non-uniformly to senescence-targeting interventions [31]. For example, direct genetic comparisons indicate that clearance of p21^WAF1/Cip1^-positive, but not p16^INK4a^-positive, SnCs prevents radiation-induced osteoporosis and marrow adiposity, consistent with unequal pathogenic weighting of marker-defined subsets [36]. More broadly, differences in effector wiring are expected to align with differences in survival dependencies and immune engagement, which can translate into state- and tissue-specific sensitivity to interventions [37]. These findings highlight the need for substate-resolved intervention design, where therapeutic strategies are tailored to the dominant effector pathway and the specific tissue pathology, rather than treating senescence as a single, uniform target.

Together, these dimensions position cellular senescence as a dynamic, condition-dependent stress-adapted state rather than a singular pathological entity. Recognizing this heterogeneity is essential for interpreting experimental findings and for informing strategies that account for differences in cell lineage, initiating drivers, and state persistence across tissues.

### 2.3 SnCs in age-related tissue dysfunction

Across organs, SnCs contribute to age-related tissue dysfunction by converting episodic stress responses into persistent, self-reinforcing failure states [9,26]. At the tissue level, a small, persistent pool of SnCs can drive dysfunction through a convergent set of effects. By distorting stem and progenitor signalling, SnCs dampen regenerative competence and bias repair toward incomplete resolution [38]. In parallel, sustained matrix turnover and pro-fibrotic cues promote maladaptive remodeling that stiffens tissue architecture and erodes functional reserve [7,26]. These changes are reinforced by sterile inflammation, as SnCs reshape immune recruitment and activation to establish chronic inflammatory niches that further impair repair and accelerate degenerative trajectories [39,40].

Causal support is strongest in settings where senescent populations can be localized to lesions, their dominant effector programs are characterized, and perturbing SnCs or senescence-associated programs improves disease-relevant endpoints. For example, in atherosclerosis, Ldlr^−/−^ plaques accumulate senescent macrophage foam cells from the earliest fatty-streak stage [41]. In advanced lesions, SnCs upregulate IL-1α, CCL2/MCP1 and MMPs, and genetic or pharmacological targeting of SnCs limits lesion growth while increasing fibrous-cap thickness, consistent with plaque stabilization [42]. In osteoarthritis, senescent chondrocytes are enriched at the articular surface and directly impair cartilage deposition in explant systems [43,44]. Genetic targeting (INK-ATTAC) or intra-articular UBX0101 reduces pain and promotes cartilage repair, accompanied by decreased MMP13, IL-6 and IL-1β and protection from age-related osteoarthritis in naturally aged mice.

Beyond these canonical examples, links between senescence and tissue dysfunction are supported by a broader yet disease-dependent evidence base, with the most convincing studies combining state-resolved mapping with organ-level functional readouts [45]. In naturally aged mice, intermittent elimination of p16^Ink4a +^ cells beginning in midlife extends median lifespan and delays multiple age-associated pathologies, consistent with a systemic contribution of SnCs burden to functional decline [36,46]. In bleomycin-injury models, interventions that target SnCs improve pulmonary function and exercise capacity even when overall fibrosis burden is only modestly changed, indicating that functional impairment can be partly uncoupled from histological fibrosis [47,48]. In the central nervous system, tauopathy models accumulate senescent-like glial populations. Interventions that target these cells attenuate tau-associated pathology and improve cognitive phenotypes, supporting the view that senescence can act upstream of tissue-level impairment in at least some neurodegenerative settings [49]. Taken together, these findings motivate disease-by-disease stratification and place functional rescue, rather than marker presence alone, at the center of assigning SnCs as drivers of age-related tissue dysfunction.

## 3 Therapeutic strategies targeting SnCs

Over the past decade, in vivo perturbation studies have shown that SnCs disproportionately impair tissue function despite their low abundance [50]. This influence is typically exerted through durable damage-response states coupled to inflammatory, matrix-remodeling, and niche-level signalling outputs that propagate dysfunction beyond the originating cells [39,40]. Therapeutic relevance is supported when an SnC-directed intervention shows on-target engagement in the intended tissue and produces concordant improvement in functional and pathology-linked endpoints within a defined intervention window, without compromising essential repair and tumour-suppressive responses [37].

**Fig. 2:**
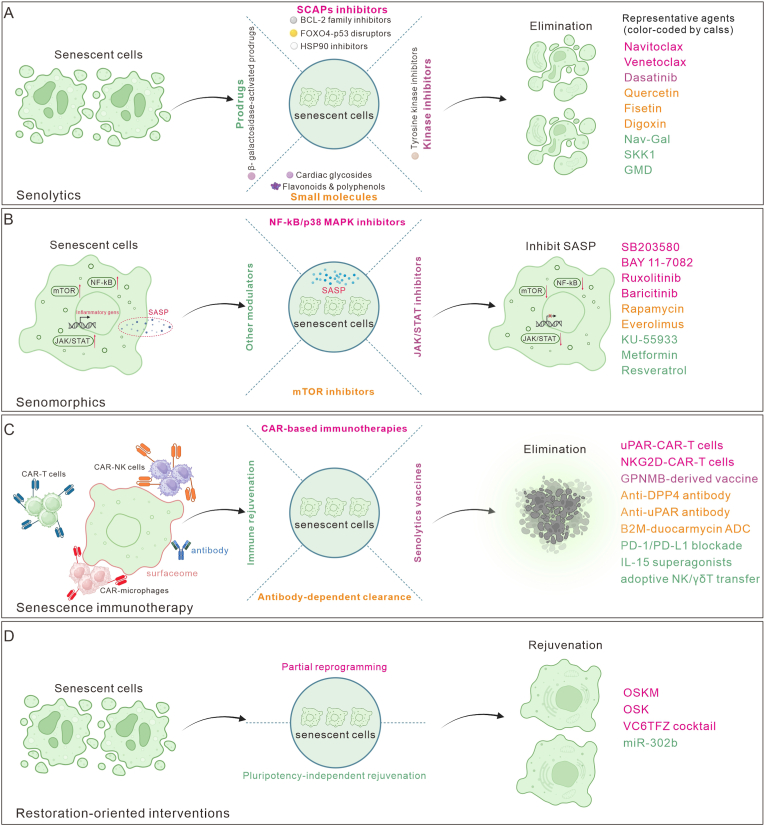
Therapeutic strategies targeting SnCs. (A) Senolytics eliminate SnCs by disrupting SCAPs or other survival pathways, with representative agents including Navitoclax, Dasatinib, and Quercetin. (B) Senomorphics suppress the SASP through inhibition of NF-κB, p38 MAPK, JAK/STAT, or mTOR signaling, using drugs such as Ruxolitinib, Rapamycin, and Metformin. (C) Immune-mediated clearance harnesses CAR-T/NK cells, antibodies, and vaccines to recognize and remove SnCs via surface antigens such as uPAR and DPP4. (D) Restoration-oriented interventions aim to recover cellular function while preserving lineage identity. Partial reprogramming transiently engages reprogramming programs (OSK/OSKM or chemical cocktails such as VC6TFZ [61]) while avoiding full pluripotency. Pluripotency-independent rejuvenation restores function by retuning senescence-stabilizing circuitry without activating pluripotency nodes (e.g., miR-302b).

Senotherapeutics encompasses a diverse array of strategies aimed at mitigating the deleterious effects of SnCs on tissue function [10,51,52]. These strategies include senolytics [53,54], which selectively target and eliminate SnCs to reduce their pathogenic influence, and senomorphics [9,51], which modulate the SASP to alleviate chronic inflammation and tissue remodeling without removing SnCs. Senescence immunotherapy leverages the immune system's capacity to recognize and clear SnCs through engineered immune cells or antibodies targeting senescence-associated markers [55,56]. Additionally, emerging restoration-oriented interventions, including partial reprogramming and pluripotency-independent rejuvenation, focus on functional recovery from senescence while preserving lineage identity. Partial reprogramming uses controlled, transient induction of pluripotency-linked factors to reset age-associated epigenetic and transcriptional states [57,58], whereas pluripotency-independent rejuvenation aims to restore function in SnCs by retuning senescence-stabilizing regulatory programs without engaging pluripotency networks [59,60]. Collectively, these senotherapeutic approaches hold significant promise for ameliorating age-related tissue dysfunction and advancing regenerative therapies (Fig. 2).

### 3.1 Senolytic strategies

Senolytic strategies utilize pharmacological agents to preferentially eliminate SnCs by targeting their heightened reliance on pro-survival and stress-resistance mechanisms [9,10] (Fig. 2A). These dependencies, often sustained through senescence-associated anti-apoptotic pathways, are less pronounced in non-senescent cells, providing a basis for selective targeting [23]. Consequently, senolytics have become a widely used approach to reduce SnC burden and are also valuable experimental tools for exploring the effects of senescent-cell clearance in vivo.

Mechanistically, senolysis can be achieved through distinct vulnerability classes, including BCL-2 family and HSP90 inhibition, disruption of FOXO4-p53 interactions, and lineage/context-specific dependencies such as dasatinib plus quercetin (D + Q) [[62], [63], [64], [65]]. Broader small-molecule families (e.g., flavonoids and cardiac glycosides) further expand the spectrum of susceptible senescent states [66,67]. Precision formats are emerging, including SA-β-galactosidase-activated prodrugs and antibody- or ligand-guided delivery to enrich exposure in disease-affected tissues [68,69].

Across models, senolytic regimens reduce senescence markers and improve tissue function in cardiometabolic, fibrotic, renal, and neurodegenerative settings, but effect size and durability depend on baseline burden, the senescent subset engaged, disease stage, and tissue exposure [10,54]. Compounds such as fisetin, navitoclax, and ARV825 show context-dependent activity [70,71] (Table 1). Early clinical studies reported feasibility and biomarker shifts with D + Q in idiopathic pulmonary fibrosis and diabetic kidney disease [72], whereas UBX0101 in knee osteoarthritis did not show efficacy [73]. Mechanism-linked toxicities (e.g., navitoclax thrombocytopenia) remain dose-limiting and motivate platelet-sparing and delivery-enabled designs with rigorous pharmacokinetics-pharmacodynamics(PK/PD) gating [74]. These results reinforce the need for engineered tissue targeting and biomarker-anchored PK/PD frameworks that quantify baseline burden, confirm on-treatment depletion in target tissues, and define an intermittent dosing cadence that is both durable and tolerable.

**Table 1:** Representative senolytic agents evaluated in preclinical models.

Compound	Disease model	Main outcomes	Limitations & negative findings
Dasatinib + Quercetin (D + Q)	• Naturally aged mice [50]	• Extended lifespan; improved muscle mass	• Limited impact on organ-level aging endpoints
	• Progeroid syndrome [23,75]	• Extended lifespan; improved skeletal health	• N/A
	• Metabolic syndrome [76]	• Reduced visceral fat; improved insulin resistance	• Less consistent benefit across model
	• Inflammatory aging model [54]	• Reduced inflammation; improved frailty index	• N/A
	• Neurodegenerative disease model [77,78]	• Reduced neuroinflammation; improved cognitive function	• Inconsistent effects on amyloid plaque burden and long-term cognitive outcomes
	• Cardiovascular aging model [79]	• Reduced plaque formation; improved endothelial function	• Minimal effects on long-term vascular health in chronic model
	• Osteoarthritis model [65]	• Improved cartilage integrity; reduced joint inflammation	• Limited benefit in advanced-stage osteoarthritis
	• Immunosenescence model [80]	• Restored immune function; enhanced vaccine responses	• No significant improvement in severe immune aging model
	• Diabetic kidney disease [81]	• Reduced renal fibrosis; improved renal function	• Variable efficacy across models; limited benefit in some settings

Fisetin	• Naturally aged mice [82]	• Improved muscle mass and physical function	• Lifespan extension remains controversial
	• Progeroid syndrome [83,84]	• Alleviated organ-specific pathology	• Minimal effects in advanced aging phenotypes
	• Metabolic syndrome [66,85]	• Reduced visceral fat; improved insulin resistance; reduced liver inflammation	• Inconsistent improvement in glucose tolerance and insulin sensitivity in long-term high-fat diet model
	• Neurodegenerative disease model [86]	• Reduced neuroinflammation; improved cognition and synaptic plasticity	• Inconsistent effects on amyloid plaque burden and durable cognitive recovery
	• Vascular disease model [82,87]	• Improved vascular function; reduced arterial stiffness; enhanced endothelial function	• Limited effects on plaque stability and long-term vascular repair
	• Kidney disease model [88,89]	• Reduced fibrosis; improved renal function and glomerular filtration	• Limited impact in advanced fibrosis and severe kidney injury

Navitoclax (ABT-263)	• Naturally aged mice [90]	• Reduced SnC burden; accelerated wound closure	• Trabecular bone loss; impaired osteoprogenitor function
	• Neurodegenerative disease model [91]	• Lowered neuroinflammation; improved spatial memory performance	• Sex-dependent effects (beneficial in males; deleterious in females)
	• Metabolic syndrome [92]	• Improved insulin sensitivity and glucose tolerance	• Thrombocytopenia
	• Atherosclerosis model [93]	• Reduced lesion burden and improved plaque stability in some settings	• In advanced disease, reduced plaque stability and increased mortality were reported
	• Cardiorespiratory disorders [94]	• Restored cardiac function	• Exacerbated pulmonary hypertension and lung injury when dosed during vulnerable phases

ABT-737	• Irradiation-induced senescence [95]	• Reduced senescence markers in irradiated tissues	• Poor oral bioavailability and low solubility limit in vivo use
	• Progeroid model [96]	• Decreased SnC burden across multi-organs	• Hematologic toxicity
	• Neurodegenerative disease model [97]	• Reduced senescent microglia and neuroinflammation; improved memory	• N/A

CB-839	• Naturally aged mice [98,99]	• Improved multi-organ function and physical performance	• N/A
	• Metabolic syndrome [100]	• Attenuated adipose senescence; improved metabolic dysfunction	• N/A

Piperlongumine	• Aortic calcification model [101]	• Reduced aortic calcium deposition	• High-dose regimens have been associated with mild, reversible renal effects
	• Stroke model [102]	• Improved EPC function; reduced infarct volume	• N/A
	• Hypoxia-associated pulmonary hypertension model [103]	• Attenuated pulmonary vascular remodeling	• N/A

PCC1	• Naturally aged mice [104,105]	• Improved late-life function and survival; improved retinal structure and function	• N/A
	• Skin fibrosis model [106]	• Improved physical performance	• N/A
	• Immune system aging model [107]	• Mitigated immune aging phenotypes	• N/A
	• Renal fibrosis model [108]	• Ameliorated renal fibrosis	• N/A
	• Diabetic wound healing model [109]	• Improved wound healing and skin quality	• N/A

ARV825	• Obesity-associated HCC model [110]	• Reduced senescent hepatic stellate cell burden	• N/A
	• Lung fibrosis model [70]	• Attenuated fibrosis	• N/A

17-DMAG	• Progeroid model [63]	• Extended healthspan; delayed multiple age-associated phenotypes	• N/A

Abbreviations: N/A, not available (not reported in the cited study) or not applicable, depending on context.

Next-generation senolytics are moving from single-node inhibition toward engineered, indication-specific killing that couples senescence features with tissue access and dominant survival dependencies [53,75]. Priorities include senescence-activated prodrugs (e.g., SA-β-gal-triggered intracellular release) and antibody/ligand-guided formats that concentrate exposure in disease-relevant niches [111]. In parallel, combination or sequential regimens are being optimized to broaden state coverage while preserving tolerability. A biomarker-anchored PK/PD framework-baseline burden, on-treatment depletion in target tissues, and cadence definition will be essential for durable benefit and a defensible safety margin.

### 3.2 Senomorphics and SASP modulation

Senomorphics mitigate SnC-driven pathology by suppressing maladaptive SASP and stress-signaling modules without depleting SnCs, aiming to reduce chronic inflammation and paracrine propagation while preserving context-dependent beneficial roles of senescence (e.g., repair and tumor suppression) [37,39] (Fig. 2B). Because SASP composition is stimulus-, tissue-, and stage-dependent, effective senomorphics require module-level rather than single-cytokine control [21,42].

SASP maintenance converges on NF-κB/C/EBP transcriptional hubs activated by DNA damage and chromatin stress [69,112,113], and on amplification circuits including p38 MAPK, mTOR, and cGAS-STING, with reinforcement via JAK-STAT and TGF-β-SMAD signaling [30,[114], [115], [116]]. In vivo, inhibition of these nodes yields tissue-level benefit (Table 2), for example JAK1/2 blockade in naturally aged mice reduced tissue inflammation and improved physical function, IKK inhibition attenuated multi-organ degeneration in Ercc1 progeroid mice, and mTORC1 inhibition limited IL-1A–NF–κB-dependent SASP and tumor-promoting activity of SnCs [29,117,118]. Human translation has largely repurposed pathway inhibitors, including metformin, rapalogs, and JAK inhibitors, but many studies have not paired clinical endpoints with tissue-level confirmation of SASP module engagement or with durability under repeated dosing [[117], [118], [119]].

**Table 2:** Representative senomorphic agents evaluated in preclinical models.

Compound	Targets	Disease model	Main outcomes	Negative & Ineffective effect
Rapamycin/Sirolimus	mTORC1/mTORC2	• Naturally aged mice [120]	• Extended lifespan; delayed senescence; enhanced tissue regeneration	• Immunosuppression; metabolic dysregulation
		• Neurodegenerative model [121]	• Improved cognition; reduced neuroinflammation	• N/A
		• Cardiovascular model [122,123]	• Improved cardiac function; reduced fibrosis	• Potential cardiovascular liabilities
		• Metabolic dysfunction model [124]	• Improved glucose metabolism; modulated lipid profiles	• May induce metabolic dysfunction with long-term administration
		• Fibrosis models [125]	• Reduced fibrosis; suppressed inflammation	• Limited efficacy in chronic fibrosis
		• Muscle aging model [126]	• Enhanced muscle regeneration; increased muscle mass	• May exacerbate sarcopenia
		• Osteoporosis model [127]	• Preserved bone density; inhibited osteoclast activity	• Bone remodeling disturbances

Ruxolitinib/Baricitinib	JAK1/2	• Naturally aged mice [117]	• Reduced systemic inflammation and SASP markers; improved physical function	• Increased susceptibility to infections
		• Metabolic syndrome [118]	• Improved glucose metabolism; reduced SASP; improved insulin sensitivity	• Incomplete correction of metabolic defects
		• Osteoarthritis model [119]	• Reduce cartilage degradation, improve joint function, decrease pro-inflammatory cytokines	• Limited cartilage repair
		• Progeria model [128]	• Reduced premature aging markers; improved skeletal function	• Potential long-term risks
		• Cardiovascular model [129]	• Reduced inflammation; improved mitochondrial function; decreased ROS; protected cardiac function and reduced fibrosis	• N/A

Metformin	AMPK/NF-κB/mTOR	• Naturally aged mice [112]	• Extended healthspan; improved metabolic function	• Did not extend maximal lifespan; effects were sex-dependent
		• Non-human primate aging [113]	• Reduced biological age markers; decelerated aging clocks; curbed chronic inflammation	• N/A
		• Osteoarthritis model [130]	• Protected cartilage; reduced inflammatory markers	• Did not fully reverse advanced cartilage degeneration
		• Intervertebral disc degeneration model [131]	• Reduced senescence and pain phenotypes; decreased local inflammation	• Did not fully restore disc homeostasis
		• Rheumatoid arthritis model [132]	• Decreased joint inflammation; modulated immune responses	• Did not fully arrest disease progression
		• Obesity-related inflammation [133]	• Reduced inflammatory SASP cytokines in adipose tissue; improved insulin sensitivity	• Limited weight loss and/or incomplete normalization of adipose SASP

p38 MAPK inhibitors	p38 MAPK	• Naturally aged mice [114]	• Alleviated aging phenotypes; improved villus structure; enhanced nutrient absorption	• Limited reversal of natural aging
		• Neurodegenerative model [115]	• Alleviates neuropathology, cognitive impairment	• N/A

NF-κB inhibitors	NF-κB pathway	• Accelerated aging model & Naturally aged mice [69]	• Reduced senescence markers; improved healthspan-related readouts	• N/A
		• Chemotherapy-induced cachexia [134]	• Mitigated senescence and inflammation	• May blunt chemotherapy-induced stress responses

BET inhibitors	BRD4, chromatin modifiers	• Atherosclerosis model [135]	• Reduced SASP expression and senescence markers	• N/A
		• Obesity model [136]	• Reduced pro-inflammatory phenotype	• N/A

Resveratrol	SIRT1/NF-κB	• Neurodegenerative model [137]	• Reduced neuroinflammation; improved cognitive function	• N/A
		• Chronic inflammation disease [138,139]	• Reduced inflammatory response	• N/A
		• Osteoarthritis model [140]	• Reduced joint inflammation	• Limited cartilage regeneration
		• Metabolic model [141]	• Improved glucose metabolism; reduced inflammatory markers	• Partial reversal of metabolic dysfunction
		• Cardiovascular aging model [142]	• Protected cardiac function	• N/A

Abbreviations: N/A, not available (not reported in the cited study) or not applicable, depending on context.

Future advancements in senomorphic strategies will focus on selectively modulating specific SASP modules to achieve tissue-specific control [37,75]. Key priorities will include decoupling chronic inflammatory signals from reparative ones, spatial and temporal restriction of SASP modulation through targeted prodrugs and delivery systems [143], and refining regimen engineering with multiplex module readouts to guide tailored combination therapies [9,75]. These developments aim to enhance the precision and effectiveness of senomorphic treatments while minimizing systemic exposure and preserving tissue function.

### 3.3 Senescence immunotherapy

Senescence immunotherapy aims to reduce SnC burden by restoring or redirecting immune surveillance and effector-mediated removal, leveraging clearance mechanisms normally executed by NK cells, macrophages, and cytotoxic T cells [56,144,145] (Fig. 2C). With aging and chronic disease, surveillance weakens, and immune cells can themselves acquire senescent phenotypes that are distinct from immunosenescence, further constraining clearance and reshaping inflammatory niches [4].

Immune-mediated targeting reflects a balance between recognition of senescence-associated stress ligands and antigens (e.g., NKG2D ligand) and local immunosuppression within senescent niches [146,147]. Preclinical proofs-of-concept include uPAR-directed CAR T cells that deplete SnCs and attenuate fibrosis in toxic and metabolic liver disease models [148], antibody-drug conjugates that deliver cytotoxic payloads to senescence-enriched targets such as B2M^+^ SnCs [9], and vaccines targeting senescence-associated surface antigens, including CD153 and GPNMB, that reduce senescence-associated stromal populations [149,150]. Clinical translation is still early, but candidate antigens can be detected in human tissues (e.g., uPAR^+^ SnCs and PD-1^+^ senescence-associated CD4^+^ T cells in obesity) [151,152], and oncology experience with engineered immune platforms provides manufacturing and toxicity-management precedent [153].

The field is moving toward surfaceome- and stress-ligand-guided antigen nomination with single-cell and spatial validation to manage heterogeneity and off-target risk [145]. Engineered effectors (logic-gated/affinity-tuned CARs, ADCs, vaccines) will need safety switches and dosing logic tailored to aged/immunocompromised hosts, where baseline immunosenescence can limit efficacy [1,154]. A translational path that demonstrates tissue-level target engagement and selective depletion, not just circulating biomarkers, will be decisive [155].

### 3.4 Restoration-oriented interventions

Restoration-oriented interventions restore youthful function by relaxing stress-stabilized constraints and rebuilding core functional capacity within lineage programs, while avoiding sustained dedifferentiation [9,60]. Unlike senolytics or senomorphics, they aim to restore core cellular capacities, including chromatin and transcriptional control, metabolic fitness, proteostasis, and stress resilience, within safety boundaries that limit identity drift and pathological expansion. Current evidence converges on two mechanistic routes(Fig. 2D). Partial reprogramming leverages the stepwise nature of reprogramming through tightly time-limited induction of pluripotency-associated factors to reset ageing-associated states without entering a stable pluripotent trajectory [58,156]. Pluripotency-independent rejuvenations, by contrast, aim to modulate senescence-enforcing nodes such as checkpoint circuitry to relax low-competence states without engaging pluripotency-associated programmes [57,59,157]. Given senescence heterogeneity, restoration claims require durable, multi-axis evidence beyond single-marker changes [7].

Partial reprogramming entails transient or cyclic induction of Yamanaka factors, most commonly OSKM (Oct4, Sox2, Klf4, c-Myc) or the reduced OSK combination (Oct4, Sox2, Klf4), under tightly constrained dose and timing, implemented as brief pulses that stop short of stable pluripotency [158,159]. Here, “partial” denotes a bounded reset of ageing-associated molecular hallmarks without complete loss of somatic identity, with safeguards that limit sustained dedifferentiation and pathological expansion [160]. Preclinical studies suggest that sufficiently constrained regimens can translate into measurable functional benefit. Cyclic OSKM improved multi-organ phenotypes and extended lifespan in progeroid LmnaG609G mice, while enhancing skeletal-muscle regeneration after injury in aged wild-type animals [159,161]. AAV-mediated OSK in retinal ganglion cells shifted DNA methylation to younger states, promoted axon regeneration, and improved vision in glaucoma models and aged mice [157]. A single transient OSKM cycle in naturally aged mice induced coordinated multi-omics shifts toward younger states across tissues [58]. Translation is entering first-in-human testing in accessible tissues with quantifiable endpoints, exemplified by an ongoing phase 1 ER-100 trial in optic neuropathies that prioritises safety, tolerability, and extended follow-up(ClinicalTrials.gov ID: NCT07290244).

Pluripotency-independent interventions, as framed by the senoreverse view [59,60], propose that stabilizing gatekeepers maintain senescence-associated dysfunction, which can be modulated in recovery-permissive states without activating pluripotency programmes. Unlike partial reprogramming, this approach targets specific constraint nodes, such as checkpoint enforcement and stress-stabilizing circuits, to re-open lineage-appropriate programmes and restore epigenetic, transcriptional, metabolic, and proteostasis control [12,162]. Given senescence heterogeneity, stringent evidence is required to distinguish true programme relaxation from selection or transient compensation, with a focus on durable concordance across state attenuation, functional restoration, and niche reprogramming, including reduced SASP and immune dysregulation [6,162]. Current evidence is primarily preclinical, with in vivo studies showing that miR-302b-mediated repression of *CDKN1A* and *CCNG2* relieves checkpoint enforcement while preserving lineage markers [59], and murine models reporting tissue-level benefits from resetting senescence-associated programmes [163]. Immediate priorities include stratifying recovery-permissive subsets, identifying actionable gatekeepers, and implementing longitudinal single-cell and functional readouts to confirm identity stability, exclude selection effects, and evaluate durability and safety [9].

Future research should focus on overcoming challenges to translate restoration-oriented interventions into clinical practice. Clinical trials are needed to assess long-term efficacy and safety, especially in regenerative tissues [156,160]. Precision delivery systems, such as tissue-targeted vectors, need further development to minimize systemic effects. Identifying and stratifying senescent subsets that are recovery-permissive will enhance patient selection and therapeutic outcomes [61,164]. Long-term monitoring through single-cell and multi-omics profiling will be essential for evaluating durability, lineage stability, and detecting potential risks [165,166]. Addressing these challenges will enable the application of restoration-oriented interventions for treating age-related diseases and enhancing tissue function.

## 4 Future directions and translational challenges

### 4.1 Defining and stratifying senescent states

As senescence-targeted modalities diversify, translation requires therapy-informing stratification rather than binary marker calls. Canonical readouts (e.g., p16^INK4a^, SA-β-gal, selected SASP factors) capture only subsets of senescent phenotypes and often blur maladaptive persistence with transient stress adaptation [12,18,41]. We therefore propose to stratify senescent states along three pragmatic axes, including persistence (maintenance after stimulus withdrawal), effector wiring (dominant inflammatory, extracellular matrix-remodeling, or IFN/ISG-like paracrine modules), and restoration responsiveness (intervention-amenable or damage-locked) [68,167]. Together, these axes provide a compact stratification framework that separates transient stress programmes from durable senescent liabilities and enables cross-tissue, cross-model comparisons.

In preclinical models, persistence can be tested by time-course designs that include stimulus withdrawal, whereas effector wiring is resolved by integrating single-cell transcriptomics with spatial profiling to localize dominant paracrine modules, complemented by secretome outputs when feasible [168,169]. Restoration responsiveness is evaluated in perturbation-withdrawal designs, requiring sustained functional rescue with preserved lineage programmes and no aberrant cycling, rather than marker shifts alone [154,170]. For clinical translation, stratification should prioritize accessible sentinel compartments complemented by longitudinal blood-based surrogates (e.g., proteins or cfDNA), each anchored to organ-level functional endpoints. This approach supports baseline stratification, on-treatment state tracking, and cross-cohort comparability, while maintaining a clear line of sight to modality selection and therapeutic development [171].

### 4.2 Safety, durability, and unintended consequences

The clinical translation of senotherapeutics hinges on long-term safety and durability, not only acute efficacy [172]. Because these interventions impinge on core systems that govern proliferation, stress adaptation, immune surveillance, and tissue maintenance, early functional gains may mask liabilities that manifest only with time, repeated exposure, or shifts in cellular state [51,134]. Risk profiles therefore differ across modalities [8,9]. Senolytics may produce off-target cytotoxicity, tissue fragility, and inflammatory rebound. Senolytics can trigger off-target cytotoxicity, compromise tissue resilience, and provoke inflammatory rebound after rapid cell loss. Senomorphics often require prolonged exposure to sustain SASP control, increasing cumulative toxicity and immunometabolic trade-offs. Senescence immunotherapy may cause collateral tissue injury and disrupt immune surveillance balance through excessive activation or imperfect antigen selectivity. Partial reprogramming can reset age-associated regulatory states but risks identity drift, aberrant proliferation, and tumorigenesis if control is imprecise. Pluripotency-independent rejuvenation aims to restore function without fate resetting, but requires rigorous safeguards against aberrant cycling, genomic instability, and tissue disruption, together with evidence of durable benefit.

Preclinical safety assessment should be designed to reveal delayed and state-dependent risks, rather than to confirm short-term efficacy [71,156]. Studies should prioritize chronic or repeated-dosing paradigms that reflect intended clinical use, incorporate aged organisms when feasible, and include washout follow-up to assess durability and rebound [2,9,146]. They should also incorporate functional challenge assays to determine whether senescence-linked protective programmes are inadvertently impaired, including cutaneous wound repair, marrow regeneration after cytotoxic stress, and immune responses to infection or vaccination [148,173]. Tumorigenic risk requires dedicated surveillance, including longitudinal multi-organ histopathology, clonal tracking, and sensitized challenge designs, supported by readouts of genomic stability and immune competence [158]. In humans, durability and safety must be assessed through longitudinal monitoring that integrates minimally invasive molecular surrogates (e.g., senescence-associated proteins, circulating nucleic acids, and epigenetic aging metrics) with organ-level functional surveillance and organismal resilience measures (e.g., frailty and performance metrics, metabolic tolerance) [15]. Trial designs should include extended follow-up, adaptive safety rules, and post-marketing registries to detect late-emerging adverse outcomes, including malignancy, impaired repair, immune dysfunction, or metabolic dysregulation [174,175].

### 4.3 From single-modality interventions to integrated senescence-targeted design

Most senescence-targeted therapies have been advanced as single-modality interventions that act on one facet of senescence biology, such as survival dependencies, inflammatory outputs, immune engagement, or epigenetic regulation [9,60]. Responses are often incomplete and tissue- or stage-dependent, reflecting differences in senescent-state persistence, functional impact, and restoration responsiveness. A recurring translational bottleneck is the weak coupling between mechanism and delivery, which limits selectivity and practical deployment [176]. Progress therefore requires an integrated design-screen-deliver framework in which target choice, screening logic, and delivery constraints are aligned to the indication.

A mechanism-delivery coupling paradigm starts from targets that enable actionable selectivity. Priority nodes include state-enriched surface antigens (eg., DPP4 or uPAR), senescent-cell survival dependencies including SCAPs, and SASP effector circuits that drive tissue dysfunction [151,177,104]. These nodes support a layered design logic that matches modality to state. Elimination is most defensible for highly deleterious, damage-locked burdens, whereas SASP control or restoration-oriented modulation is favored when preserving tissue integrity is paramount and functional rescue is plausible [117,178]. In this architecture, senolysis, senomorphic control, and restoration-oriented interventions operate as complementary options rather than competing concepts.

Delivery must be equally strategic because selectivity is ultimately enforced by exposure. Tissue-directed carriers, organ-restricted systems, and BBB-competent platforms should be chosen to match mechanism and risk [37,179]. Practical options include conditionally activated prodrugs to limit systemic toxicity, ADC or ligand-guided payloads to exploit surface antigens, and modalities such as PROTACs, vaccines, or engineered cell therapies when durable or localized action is required [149,180]. Integrated design further depends on mechanism-driven screening that measures state transitions and functional rescue, incorporates combinatorial and intermittent regimens, and links PK/PD to predefined burden or imaging thresholds as go/no-go decision gates.

## Conflict of interest

The authors have no financial or ethical conflicts of interest to declare.
